# Development of a shutterless continuous rotation method using an X-ray CMOS detector for protein crystallography

**DOI:** 10.1107/S0021889809042277

**Published:** 2009-11-17

**Authors:** Kazuya Hasegawa, Kunio Hirata, Tetsuya Shimizu, Nobutaka Shimizu, Takaaki Hikima, Seiki Baba, Takashi Kumasaka, Masaki Yamamoto

**Affiliations:** a SPring-8/JASRI, 1-1-1 Kouto, Sayo-cho, Sayo-gun, Hyogo 679-5198, Japan; b RIKEN SPring-8 Center, 1-1-1 Kouto, Sayo-cho, Sayo-gun, Hyogo 679-5148, Japan

**Keywords:** protein crystallography, shutterless continuous rotation method, X-ray CMOS detectors, X-ray wavelength capabilities

## Abstract

A new shutterless continuous rotation method using an X-ray complementary metal-oxide semiconductor (CMOS) detector has been developed for high-speed, precise data collection in protein crystallography. The principle of operation and the basic performance of the X-ray CMOS detector (Hamamatsu Photonics KK C10158DK) have been shown to be appropriate to the shutterless continuous rotation method. The data quality of the continuous rotation method is comparable to that of the conventional oscillation method using a CCD detector and, furthermore, the combination with fine ϕ slicing improves the data accuracy without increasing the data-collection time. The new method is more sensitive to diffraction intensity because of the narrow dynamic range of the CMOS detector. However, the strong diffraction spots were found to be precisely measured by recording them on successive multiple images by selecting an adequate rotation step. The new method has been used to successfully determine three protein structures by multi- and single-wavelength anomalous diffraction phasing and has thereby been proved applicable in protein crystallography. The apparatus and method may become a powerful tool at synchrotron protein crystallography beamlines with important potential across a wide range of X-ray wavelengths.

## Abstract 2

A shutterless continuous rotation method using an X-ray complementary metal-oxide semiconductor (CMOS) detector has been developed for high-speed, precise data collection in protein crystallography. The new method and detector were applied to the structure determination of three proteins by multi- and single-wavelength anomalous diffraction phasing and have thereby been proved to be applicable in protein crystallography.

## 1. Introduction   

The oscillation method with an X-ray CCD area detector has been widely used in the past decade or so at synchrotron beamlines for protein crystallography to collect diffraction data sets. The combination of highly intense X-ray beams and fast-readout CCD detectors has enhanced the data-collection speed and increased the throughput of structure determination.

However, there is still potential to improve the oscillation method for high-quality data collection. The most effective improvement is the utilization of fine ϕ slicing, because the background scattering from air and solvent around a crystal sample decreases and the signal-to-noise ratio (S/N) of the diffraction intensities is improved (Pflugrath, 1999 ▶). Snell *et al.* (1995 ▶) probed the limits of how small the mosaicity could be for a protein crystal and thereby the S/N at the highest-resolution weak-reflection part of the diffraction pattern. They highlighted the importance of fine ϕ slicing, because these weak reflections would be swamped with background if coarse ϕ slicing was used. However, there are difficulties in utilizing a small rotation step because the number of images and partially recorded reflections increase as the oscillation step gets smaller. The main difficulty is improper synchronization of the shutter and the goniometer, which causes a deterioration in the data accuracy of partially recorded diffractions. Another difficulty is the overhead time between successive exposures. It consists of two time domains of reading out images and accelerating and decelerating the goniometer. The overhead time is sometimes comparable to or longer than the exposure time of one image, resulting in a lot of beam time being wasted.

In order to achieve efficient data collection and overcome these limitations, a new data-collection method, called the shutterless continuous rotation method, has been proposed (Ito *et al.*, 2000 ▶, 2004 ▶; Brönnimann *et al.*, 2003 ▶). In this method, once diffraction data collection starts, the X-ray shutter is kept open and the goniometer is rotated continuously with a constant angular speed. The X-ray detector outputs diffraction images continuously with a constant frame rate during data collection. The rotation step per image is defined by the ratio of the angular speed of the goniometer and the frame rate.

The essential features of the X-ray detector for the continuous rotation method can be defined as fast and continuous readout. A full frame transfer CCD as used in protein crystallography cannot be continuously read out and is thus unsuitable for this purpose. Therefore, several detector types for this purpose have been developed. At the Photon Factory in Japan, Ito *et al.* (2007 ▶) developed a new X-ray CCD detector using a frame transfer CCD and employed the continuous rotation method. At the Paul Scherrer Institut in Switzerland, a hybrid pixel detector, ‘PILATUS’, with a readout time of several milliseconds (3.6 ms for PILATUS 6M; DECTRIS, Baden, Switzerland) has been developed, and the continuous rotation method has been examined at the Swiss Light Source (Brönnimann *et al.*, 2003 ▶, 2006 ▶; Hülsen *et al.*, 2006 ▶). Miyoshi *et al.* (2008 ▶) have developed an X-ray high-gain avalanche rushing amorphous photoconductor–field emitter array (HARP–FEA) detector system for high-throughput protein crystallography and recorded diffraction images from protein crystals using the continuous rotation method at the Photon Factory in Japan.

Here we introduce the X-ray complementary metal-oxide semiconductor (CMOS) detector, model type Hamamatsu Photonics KK C10158DK (Hamamatsu, Japan), as a new promising candidate for the continuous rotation method with X-rays. The CMOS detector is a two-dimensional X-ray detector composed of a phosphor and a CMOS image sensor, and can be used for the continuous rotation method because it can read out images continuously with a high frame rate; also, the dead time due to readout is negligible. The CMOS detector was originally developed for real-time imaging, such as nondestructive inspection or radiography. The CMOS detector has been improved for use in protein crystallography in collaboration with Hamamatsu Photonics KK (Yagi *et al.*, 2004 ▶; Yagi & Inoue, 2007 ▶). The basic performance of the CMOS detector and the application results for shutterless continuous data collection are reported here.

## 2. Method   

### 2.1. CMOS detector C10158DK   

The Hamamatsu Photonics C10158DK detector (Table 1 ▶) adopts the active-pixel-type CMOS image sensor, which is equipped with amplifiers in each pixel so that the output signal is resistant to readout noise (Yagi & Inoue, 2007 ▶). The phosphor layer, composed of a 150 µm-thick columnar CsI(Tl) crystal, is directly deposited on the CMOS image sensor for improvement of spatial resolution. Since there is no high-density substrate at the surface of the phosphor layer, the sensitivity for long-wavelength X-rays has been improved (Yagi *et al.*, 2004 ▶). For short-wavelength X-rays, a 150 µm-thick CsI(Tl) is very advantageous because of its high absorption (*e.g.* 97% for a wavelength of 0.8 Å).

The CMOS sensor of C10158DK is so large that the active area of 117.6 × 117.6 mm can be covered by a single CMOS sensor. This enables direct coupling with phosphor and the CMOS image sensor without demagnification, and means it is free from issues such as attenuation of fluorescence intensity and distortion of diffraction images caused by demagnification devices such as a fibre optics taper.

The C10158DK is not equipped with a cooling system to reduce dark-current noise, because the active-pixel CMOS image sensor with directly deposited phosphor would be expected to increase the S/N sufficiently and a short exposure time would be expected to decrease the influence of the dark current. This simple detector architecture makes it compact and reduces the cost of manufacturing.

The readout sequence of the CMOS sensor in C10158DK is as follows. First, a horizontal pixel array is simultaneously processed in 14 µs. In this step, all signals in the horizontal pixel array are converted into a voltage signal and transferred to the hold-capacitors located at one edge of the sensor. Then, the signals in the hold-capacitors are sequentially read out in 140 µs. The photodiodes can immediately accumulate the signals following completion of the signal transfer to the hold-capacitors. Therefore, the effective dead time is only 14 µs, which is negligible compared with typical, current exposure times per diffraction image. The readout of horizontal lines proceeds sequentially from the first to the last line and there is a time lag of 140 µs between neighbouring lines. The readout of the entire image is completed in 0.333 s, and a maximum frame rate of 3 frames s^−1^ is available with this detector.

### 2.2. Experimental setup   

Experiments for evaluation of the new shutterless continuous rotation method using the X-ray CMOS detector were performed at SPring-8 (Hyogo, Japan). The linearity and uniformity of the detector were examined at the SPring-8 beamline BL41XU to make use of the highly intense X-rays from the undulator. A comparison between the conventional oscillation method using a Quantum 210 CCD detector (Q210; ADSC, California, USA) and the continuous rotation method using the CMOS detector was conducted at the SPring-8 beamline BL38B1. Data collection for the structure determination of proteins using multi- (MAD) and single-wavelength anomalous diffraction (SAD) phasing was carried out at the SPring-8 beamline BL44B2.

BL41XU is an undulator beamline, which provides intense X-rays with a photon flux of 1 × 10^12^ photons s^−1^ at 1 Å with a beam size of 50 × 50 µm (FWHM). BL38B1 is a bending-magnet beamline, with a photon flux of 8 × 10^10^ photons s^−1^ at 1 Å and 9 × 10^10^ photons s^−1^ at 0.8 Å with a beam size of 210 × 140 µm (FWHM). BL44B2 is also a bending-magnet beamline, with a photon flux of 1 × 10^11^ photons s^−1^ at 1 Å with a beam size of 200 × 200 µm (FWHM).

The detector was operated at room temperature (297 K) with the following setup. Digitized diffraction images were transferred to the detector control PC and captured by a frame grabber board (IMAQ PCI-1424; National Instruments, Texas, USA). The frame rate of the CMOS detector was controlled by an external trigger pulse generated by a function generator (DG535; Stanford Research, California, USA).

### 2.3. Evaluation of the basic performance of the CMOS detector   

The dark current and readout noise were evaluated by measuring successive blank images (100 in all) recorded at various accumulation times. The linearity of the detector was verified using the powder diffraction pattern of CaSO_4_·2H_2_O recorded with various photon fluxes of the incident 1 Å wavelength X-rays. The incident X-ray intensity was attenuated stepwise by changing the thickness of an aluminium attenuator with an exposure time of 0.5 s. The photon flux of the incident beam was measured using an Si PIN photodiode at each step. For flat-field measurement, the detector was uniformly illuminated with fluorescence X-rays of 0.96 Å wavelength from a saturated NaBr solution that was sealed in a glass capillary. Flat-field images were recorded with 1 s exposure time. The detector was placed at 460 mm distance and 90° to the incident-beam direction *i.e.* perpendicular.

### 2.4. Comparison with the conventional oscillation method using a CCD detector   

The performance of the continuous rotation method using the CMOS detector was compared with the conventional oscillation method using a CCD detector, the Q210. Here we focused on the difference of the dynamic range and sensitivity in both detectors; thus the experiment was performed with various intensities and wavelengths of incident X-rays under the following three conditions: (i) a low intensity suitable for the CMOS detector at 1 Å X-ray wavelength, (ii) a high intensity suitable for the CCD detector at 1 Å X-ray wavelength and (iii) a low intensity suitable for the CMOS detector at 0.8 Å X-ray wavelength. The detector distance in all data sets was 75 mm. The Q210 was operated in nonbinning mode to set the pixel size comparable to that of the CMOS detector. All data sets were processed with the *XDS*/*XSCALE* software package (Kabsch, 1993 ▶, 2001 ▶) except where stated otherwise.

#### 2.4.1. A relatively low intensity condition suitable for the CMOS detector at 1 Å X-ray wavelength   

Three data sets were collected from one hen egg white lysozyme crystal in the tetragonal form using a crystal size of 135 × 80 × 50 µm under conditions where the maximum intensity of diffraction images was comparable to the saturation level of the CMOS detector. The first and the third data sets were collected by the CCD detector and the second data set was collected by the CMOS detector. The third data set was a reference to check the possibility of X-radiation damage. The rotation step and exposure time were 0.5° frame^−1^ and 2 s frame^−1^, respectively, and a total of 180 images were recorded for all data sets. The X-ray wavelength was 1 Å, and an aluminium attenuator of 400 µm thickness was used.

#### 2.4.2. A relatively high intensity condition suitable for the CCD detector at 1 Å X-ray wavelength   

The comparison was also performed under conditions where the maximum intensity of diffraction images was comparable to the saturation level of the CCD detector. The size of the lysozyme crystal (185 × 135 × 95 µm) was larger than that in §2.4.1 and the thickness of the attenuator was set to 200 µm so that the diffraction intensity increased. The first and the second data sets were collected with the same exposure time and rotation step as in §2.4.1. In the third data set, 900 diffraction images were collected using the CMOS detector with a 0.1° frame^−1^ step and 0.4 s frame^−1^ exposure. A reference data set with identical conditions to the first data set was finally collected with the CCD detector. The total rotation range and the total exposure time were identical for all data sets.

#### 2.4.3. A relatively low intensity condition suitable for the CMOS detector at 0.8 Å X-ray wavelength   

A comparison was also carried out at a wavelength of 0.8 Å under conditions where the maximum intensity of diffraction images was comparable to the saturation level of the CMOS detector. Three data sets were collected from one lysozyme crystal of size 145 × 100 × 50 µm using the same procedure as that described in §2.4.1. An aluminium attenuator of 400 µm thickness was used.

### 2.5. Evaluation of the continuous rotation method with fine ϕ slicing   

Two series of data sets were collected in order to (i) compare the influence of the slicing rotation step on data accuracy between the continuous rotation method using the CMOS detector and the oscillation method using the CCD detector, and (ii) examine the performance of the continuous rotation method using the CMOS detector combined with the fine ϕ slicing data collection.

#### 2.5.1. Comparison of slicing rotation steps between two data-collection methods   

Seven data sets at 1 Å X-ray wavelength were collected from one lysozyme crystal of size 115 × 105 × 50 µm. The first three data sets were successively collected using the CCD detector with oscillation steps of 1.0, 0.5 and 0.25° frame^−1^, and exposure times of 4, 2 and 1 s frame^−1^, respectively. The next three data sets were collected by the continuous rotation method using the CMOS detector. The rotation steps and exposure times per frame were the same as those of the CCD data. The last data set was collected with the same conditions as the first data set for reference. The total rotation range of 90° and total exposure time of 360 s were identical for all data sets. An attenuator of 600 µm-thickness aluminium was used in order to collect seven data sets from one crystal without significant radiation damage. The detector distance for all data sets was 75 mm. The Q210 was operated in nonbinning mode.

#### 2.5.2. Effects of fine ϕ slicing   

Four data sets were sequentially collected by the CMOS detector from one lysozyme crystal of size 140 × 95 × 50 µm. The rotation steps were 0.1, 0.2, 0.4 and 1.0° frame^−1^, and the exposure times 0.5, 1.0, 2.0 and 5.0 s frame^−1^. A fifth data set as the reference was finally collected with the same conditions as the first data set. The total rotation was 90° and the total exposure time was 450 s for all data sets. A 0.8 Å X-ray wavelength was used to record high-resolution diffraction data and a 400 µm-thickness aluminium attenuator was used. The detector distance was set at 70 mm.

### 2.6. Structure determination by the continuous rotation method   

Diffraction data were collected using three protein crystals to evaluate the performance of the shutterless continuous rotation method using the CMOS detector. One was a mercury derivative of a hen egg white lysozyme crystal in the tetragonal form, which was prepared by soaking crystals in 5 m*M* *p*-chloromercuribenzene sulfonic acid solution for 10 h (Blake *et al.*, 1965 ▶). The other two proteins were a putative cobalt transport ATP-binding protein ST1066 from *Sulfolobus tokodaii* [NCBI accession No. NP_376986, Protein Data Bank (PDB) code 2PJZ] and a thioredoxin-like protein TTHA0593 from *Thermus thermophilus* HB8 (NCBI accession No. YP_143859, PDB code 2CVB). The mercury derivative of hen egg white lysozyme and two selenomethionine proteins ST1066 and TTHA0593 were used for MAD and SAD experiments.

## 3. Results   

### 3.1. Basic performance of C10158DK   

The basic performance of the detector such as dark current, readout noise, linearity and non-uniformity was evaluated first, because this evaluation was necessary for diffraction image correction.

#### 3.1.1. Dark current and readout noise   

**Figure 1:**
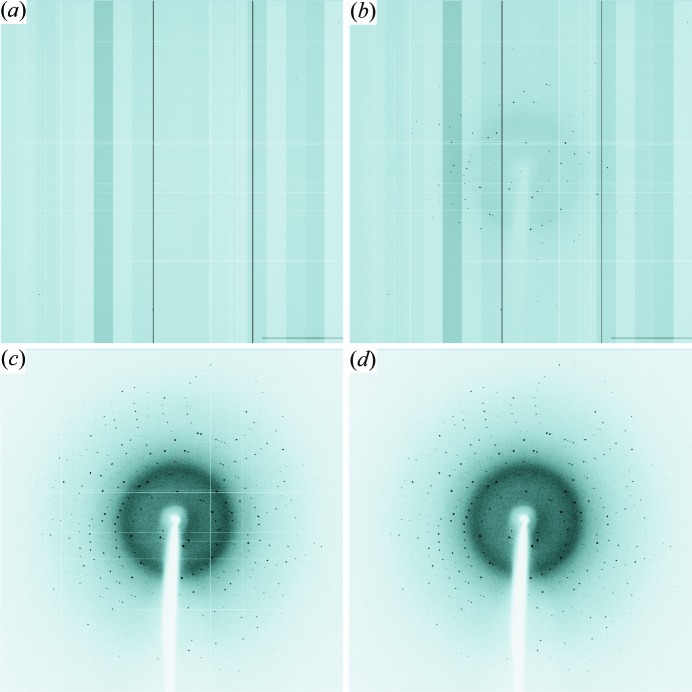
(*a*) Dark-current image recorded using C10158DK with a 1 s accumulation time. (*b*) Raw diffraction image of hen egg white lysozyme recorded with a 1 s exposure. (*c*) Diffraction image obtained by subtracting the dark current from the raw diffraction image shown in (*b*). (*d*) Diffraction image obtained by applying a defect line correction to the image in (*c*).

Figs. 1 ▶(*a*) and 1 ▶(*b*) show a dark-current image and a raw diffraction image of a hen egg white lysozyme crystal recorded with a 1 s exposure time and a 1 s accumulation time, respectively. The vertical stripe pattern on the images is caused by the output offset between the 18-channel amplifier arrays located at the bottom edge of the sensor. About ten defect lines were observed in the image as white or black lines running horizontally or vertically.

**Figure 2:**
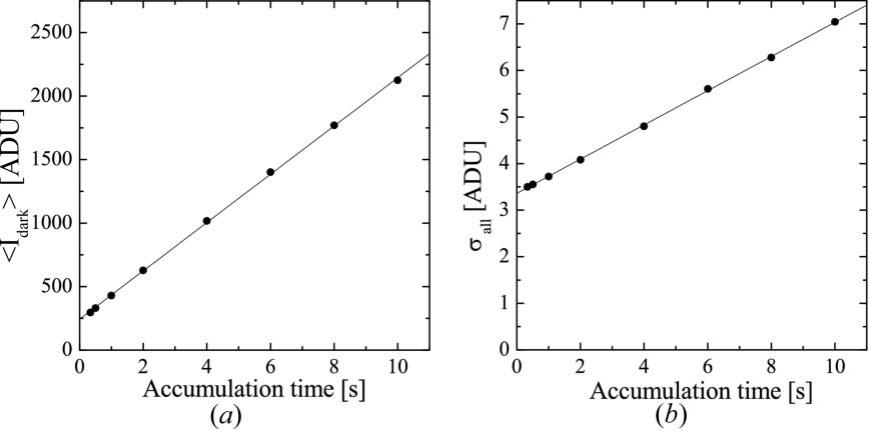
(*a*) Average dark current (〈*I*_dark_〉) plotted against accumulation time. (*b*) Total detector noise (σ_all_), which is composed of the readout noise (σ_readout_) and fluctuation of dark current (σ_dark_), also plotted against accumulation time.

The mean dark current (〈*I*_dark_〉) calculated by averaging the dark levels over all pixels is plotted against accumulation time in Fig. 2 ▶(*a*). The defect pixels were excluded from the calculation. The 〈*I*_dark_〉 value increased by 190 analog-to-digital converter units (ADU) per second in proportion to the accumulation time. Extrapolating 〈*I*_dark_〉 to 0 s accumulation time led to the offset of 243 ADU. The 〈*I*_dark_〉 at 1 s accumulation was 430 ADU, whereas it became 2125 ADU at 10 s accumulation, which is one-eighth of the saturation level (14 bits).

The fluctuations of dark current (σ_dark_) and the readout noise (σ_readout_) were estimated as follows. First, the standard deviation of the dark current in the 100 successively recorded dark-current images was calculated at each pixel. Then, the standard deviations were averaged over all pixels (σ_all_), which is the total detector noise composed of σ_dark_ and σ_readout_. Fig. 2 ▶(*b*) is a plot of σ_all_ against accumulation time, which showed that σ_all_ increased in proportion to the accumulation time. The increase of σ_all_ was due to an increase of σ_dark_, because σ_readout_ is independent of the accumulation time. Therefore, extrapolation of σ_all_ to 0 s accumulation time yielded a σ_readout_ estimate of 3.37 ± 0.27 ADU (mean ± standard deviation). σ_dark_ is estimated as (σ_all_^2^ − σ_readout_^2^)^1/2^ based on the law of propagation of errors. σ_dark_ at 1 s accumulation is calculated as 1.6 ADU.

#### 3.1.2. Linearity and uniformity   

**Figure 3:**
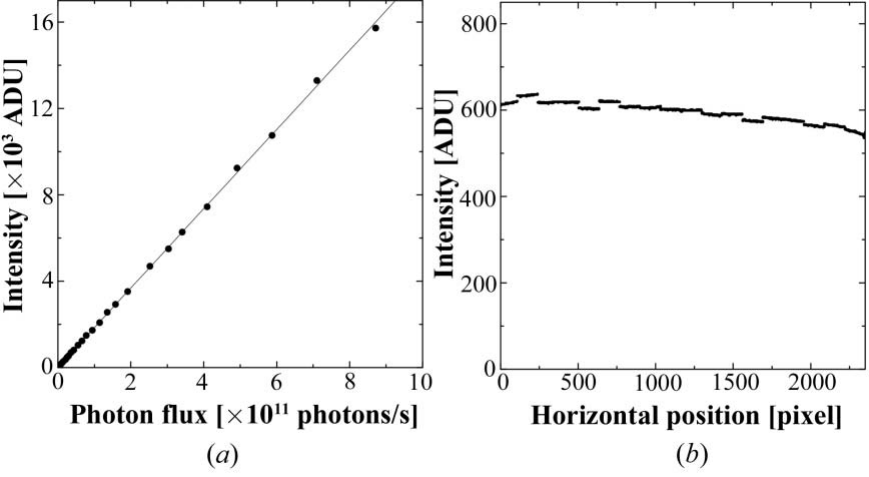
(*a*) Linearity of C10158DK. After averaging five diffraction images of a CaSO_4_·2H_2_O powder pattern to improve S/N, the dark current was subtracted. The intensity of one pixel for the 020 reflection was plotted against the photon flux of the incident X-ray beam. (*b*) Horizontal profile of the flat-field image. After averaging 100 raw images, the dark-current image was subtracted. Average profiles of the central 264 rows are shown. The incident X-rays travelled from the left side to the right side in this figure. A gradual change in intensity with the pixel position can be explained by the absorption of the incident X-rays and fluorescence X-rays by the NaBr solution; the fluorescence X-rays emitted from different points on the incident-beam path decrease gradually along with the incident beam because the incident X-ray is reduced by absorption. In addition, the intensity of the fluorescence X-ray is asymmetrically attenuated by the absorption and has a different intensity depending on the direction, unless it was emitted from the centre of the capillary. Therefore, convolution of the intensity of fluorescent X-rays emitted from multiple points generates an asymmetrically gradually changing profile. Calculation of the intensity profile considering the effect of absorption generates almost the same profile (data not shown).

The linearity of the detector was evaluated by plotting the diffraction intensity for a CaSO_4_·2H_2_O powder sample against the photon flux of the incident X-ray beam (Fig. 3 ▶ *a*). The minimum and maximum intensities were 4 and 15 725 ADU, respectively, and the *R* ^2^ value of the regression line was 0.9998. This result indicates that the linearity of C10158DK is maintained over the output data range of 14 bits, or 16 383 ADU.

The horizontal profile of the flat-field image is shown in Fig. 3 ▶(*b*). It shows a small difference in sensitivity among the 18 readout segments. The maximum difference between the neighbouring segments was 3% of the average intensity of these segments. The profile also shows a gradual non-uniformity along the horizontal position. In order to verify whether the gradual change was due to an uneven response of the detector or the non-uniformity of the flat-field X-ray, five flat-field images were recorded by changing the horizontal position of the detector by 30 mm. Superposition of the horizontal profiles of these images with a horizontal offset of 30 mm (or 600 pixels) per image showed that the profiles were well fitted to each other. This indicated that the gradual change might be caused by the non-uniformity of the flat-field X-ray illumination.

#### 3.1.3. Image correction   

**Figure 4:**
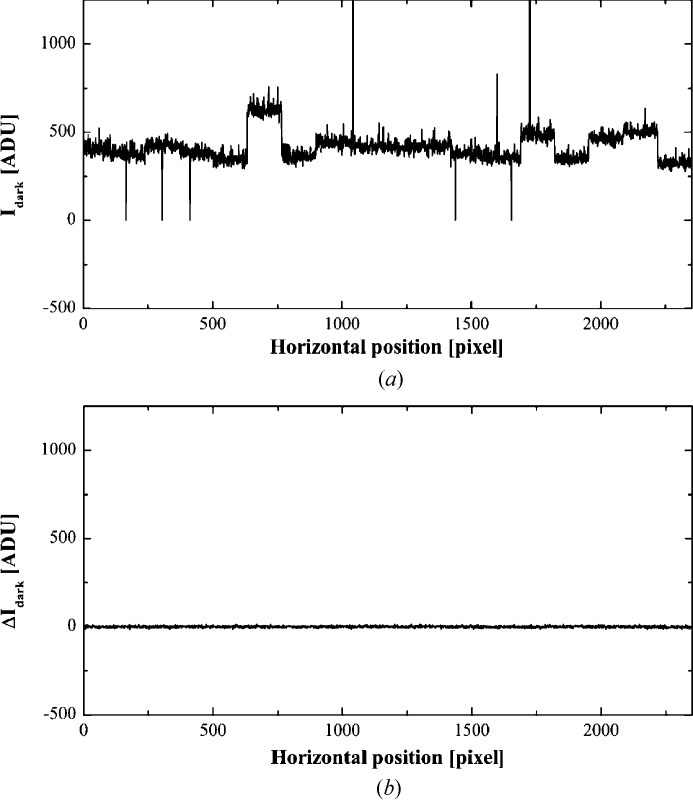
(*a*) Intensity profile of the 1000th row of a dark-current image recorded with an accumulation time of 1 s. (*b*) A difference profile between two dark-current images recorded with a 1 s accumulation time. The standard deviation of the difference profile is 5.1 ADU which is comparable to convolution of the σ_all_ values of the two images.

The use of a short exposure time gives a small 〈*I*_dark_〉 and maximizes the effective dynamic range. Moreover, the fluctuation of dark current is so small that the effect of dark current can be corrected only by subtracting a pre-recorded dark-current image. The effectiveness of dark-current subtraction is shown in Fig. 4 ▶, where a horizontal profile of a dark-current image and a difference intensity profile of two dark-current images are compared. The dark-current images for correction were recorded just before starting a series of data-collection images with the same exposure time. The corrected diffraction image in Fig. 1 ▶(*c*) was obtained by applying dark-current corrections to the raw diffraction image in Fig. 1 ▶(*b*). The diffraction spots that were obscure in the raw image were clearly observed. The readout noise and fluctuation of the dark-current image were reduced by averaging multiple dark-current images.

Another treatment was a defect line correction. Some defect pixels had a different output offset and different conversion gains from those of normal pixels, and the other defect pixels output no signal. The intensity of all of these defect pixels was padded with the average value of the neighbouring pixels. The diffraction image in Fig. 1 ▶(*d*) was obtained by applying the defect line correction to the diffraction image in Fig. 1 ▶(*c*).

### 3.2. Comparison with the conventional oscillation method using the CCD detector   

#### 3.2.1. A relatively low intensity condition suitable for the CMOS detector at 1 Å X-ray wavelength   

**Figure 5:**
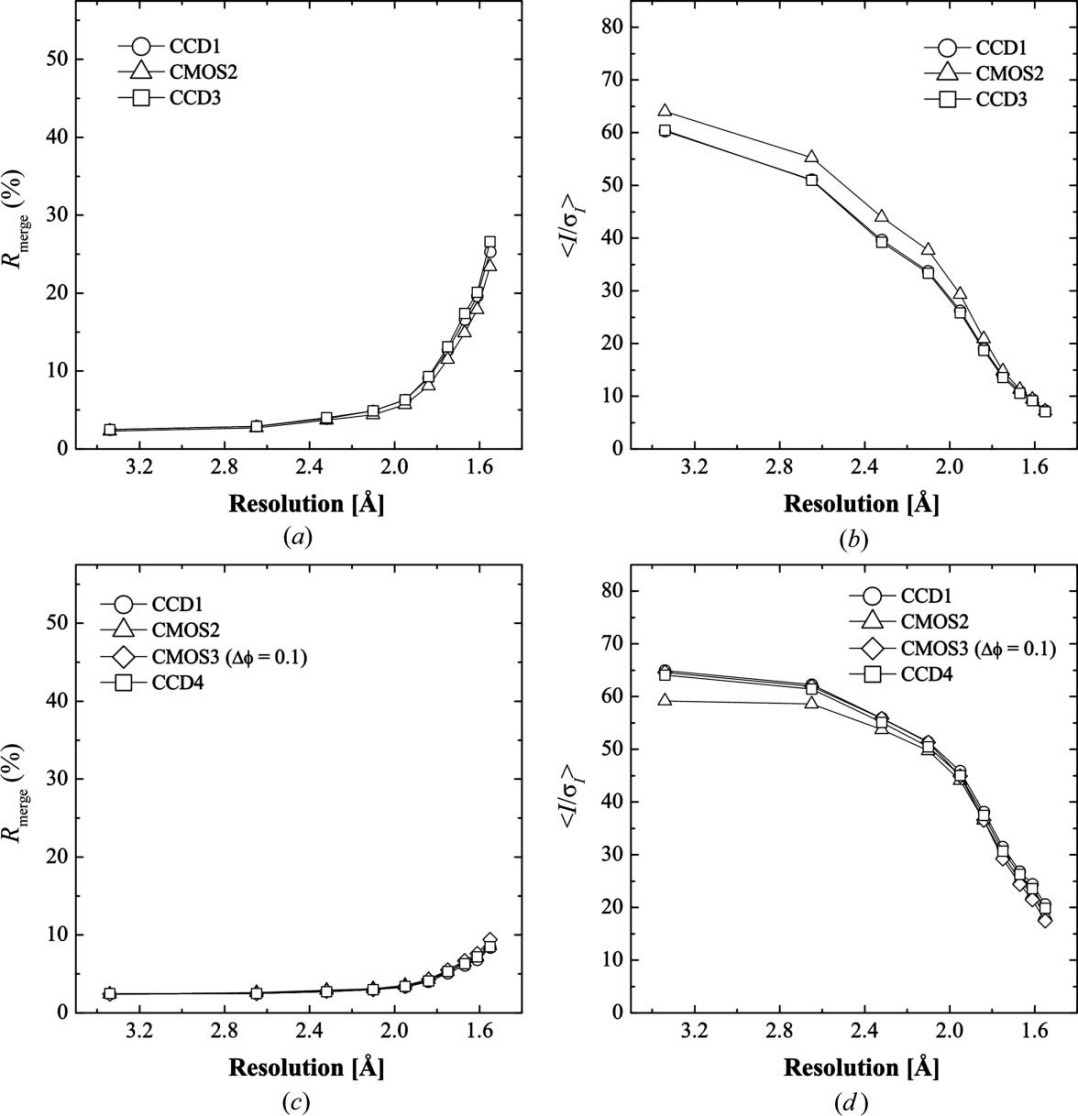
*R*_merge_ and 〈*I*/σ(*I*)〉 plotted against resolution shells. Statistics of CCD and CMOS data are plotted as open circles and open triangles, respectively. Open rectangles represent the CCD data collected to examine the effect of radiation damage. The open diamonds shown in (*c*) and (*d*) represent CMOS data collected with a rotation step of 0.1° frame^−1^; the other data were collected with a rotation step of 0.5° frame^−1^. The differences in the measurement conditions in (*a*), (*b*) and (*c*), (*d*) are the size of the crystal and the attenuator thickness; the diffraction intensity of data (*c*), (*d*) is stronger than that of data (*a*), (*b*). The data-set names in the graph legends are composed of the detector type, CCD or CMOS, and the data-collection sequence – for example, CMOS2 is the second data set recorded with the CMOS detector. This nomenclature is also used in Figs. 6 ▶–8 ▶ ▶.

*R*_merge_ and 〈*I*/σ(*I*)〉 are plotted against resolution shell in Figs. 5 ▶(*a*) and 5 ▶(*b*). The statistics of the first and the third data sets collected by the CCD detector showed almost the same results, which means that the sample radiation damage was negligible. *R*_merge_ and 〈*I*/σ(*I*)〉 of the CMOS data were slightly better than those for the CCD data.

#### 3.2.2. A relatively high intensity condition suitable for the CCD detector at 1 Å X-ray wavelength   

In order to evaluate the influence of the relatively narrow dynamic range of the CMOS detector, the data accuracy was examined under conditions where the maximum intensity of the images was comparable to the saturation level of the CCD detector.

The completeness of the CMOS data collected with 0.5° frame^−1^ was 92.5% at the lowest-resolution shell and that of the CCD data was 98.7%, indicating that there were a number of saturated reflections in the CMOS data. Figs. 5 ▶(*c*) and 5 ▶(*d*) show the comparison of the data statistics. The *R*_merge_ values of the CCD data and the CMOS data are comparable, unlike the result in §3.2.1. The 〈*I*/σ(*I*)〉 of the CMOS data collected with 0.5° frame^−1^ were lowest among all the data sets in the resolution range of 40–2.0 Å, because strong reflections might be rejected as saturated. The completeness at the lowest shell of the CMOS data collected with 0.1° frame^−1^ was successfully recovered to 96.6%, because the strong reflections were divided into multiple images.

#### 3.2.3. A relatively low intensity condition suitable for the CMOS detector at 0.8 Å X-ray wavelength   

**Figure 6:**
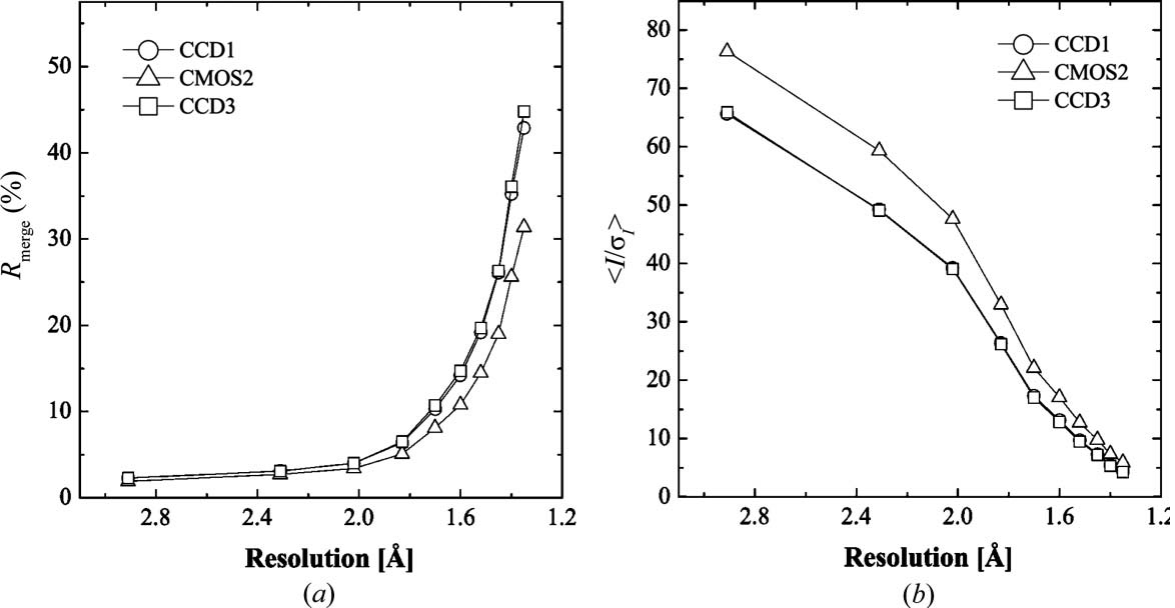
*R*_merge_ and 〈*I*/σ(*I*)〉 plotted against resolution shell. Statistics of CCD and CMOS data are plotted as open circles and open triangles, respectively. Open rectangles represent CCD data collected to examine the effect of sample radiation damage.

The comparison was also made using a 0.8 Å X-ray wavelength. *R*_merge_ and 〈*I*/σ(*I*)〉 are compared in Figs. 6 ▶(*a*) and 6 ▶(*b*), which show that the data accuracy in the high-resolution shells of the CMOS data was better than that of the CCD data.

### 3.3. Evaluation of the continuous rotation method with fine ϕ slicing   

#### 3.3.1. Comparison of slicing rotation step between the two data-collection methods   

**Figure 7:**
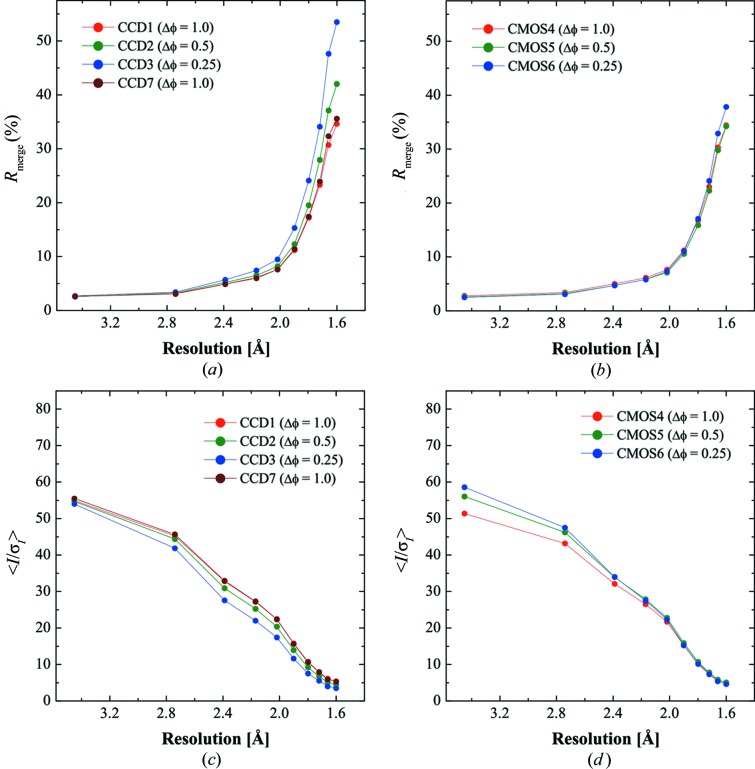
The effect of rotation (oscillation) step volume compared for the CCD and the CMOS data. (*a*) and (*b*) show *R*_merge_ *versus* resolution plots for the CCD and the CMOS data, respectively. (*c*) and (*d*) are 〈*I*/σ(*I*)〉 *versus* resolution plots of the CCD and the CMOS data, respectively. Red, green and blue represent data sets collected with the rotation steps of 1.0, 0.5 and 0.25° frame^−1^, respectively. Brown in (*a*), (*c*) corresponds to the last data set collected to examine the effect of sample radiation damage.

Figs. 7 ▶(*a*) and 7 ▶(*b*) show *R*_merge_ *versus* resolution plots that compare statistics among data sets collected with 1.0, 0.5 and 0.25° frame^−1^ steps. The *R*_merge_ of the CCD data at the highest-resolution shells increased from 34.6 to 53.5% as the oscillation step got smaller, as shown in Fig. 7 ▶(*a*). The *R*_merge_ values of the CMOS data collected with 1.0, 0.5 and 0.25° frame^−1^ were 34.4, 34.2 and 37.8%, respectively, in the highest-resolution shell (Fig. 7 ▶ *b*), and are almost comparable to those of the CCD data collected with 1° frame^−1^. The CCD data were also processed using *MOSFLM* (Leslie, 1992 ▶) and *SCALA* (Evans, 1993 ▶) in order to compare the *R*_merge_ of fully recorded reflections among these data sets (Table 2 ▶). This shows that the redundancy-independent *R*_merge_ values for fully recorded reflections are comparable between CCD data, indicating that the decrease of CCD data accuracy was due to the influence of partially recorded reflections.

Figs. 7 ▶(*c*) and 7 ▶(*d*) give the 〈*I*/σ(*I*)〉 *versus* resolution plots of the CCD data and CMOS data, respectively. Fig. 7 ▶(*c*) shows that 〈*I*/σ(*I*)〉 of the CCD data became smaller as the rotation step decreased in all resolution shells. This result means that the smaller rotation step causes a deterioration in the S/N of the diffraction intensities. On the other hand, 〈*I*/σ(*I*)〉 of the CMOS data became larger as the rotation steps got smaller up to 2 Å resolution, and had almost the same value at higher than 2 Å resolution. This means that the S/N of strong reflections up to 2 Å resolution was improved by more finely slicing the rotation step. However, there was no improvement for weak reflections above 2 Å.

#### 3.3.2. Effects of fine ϕ slicing   

As described above, the finer slicing of the rotation step improved the data accuracy of the CMOS data for the strong reflections in the low-resolution shells. This indicates that the use of a smaller rotation step would improve data accuracy further if the diffraction intensity was stronger. In order to verify this, experiments using rotation steps of 0.1, 0.2, 0.4 and 1.0° frame^−1^ were performed under conditions that provided a stronger diffraction intensity: there were a couple of saturated spots per image for the 0.1° frame^−1^ data set, and about 25 saturated spots per one image of 1.0° frame^−1^ data sets.

**Figure 8:**
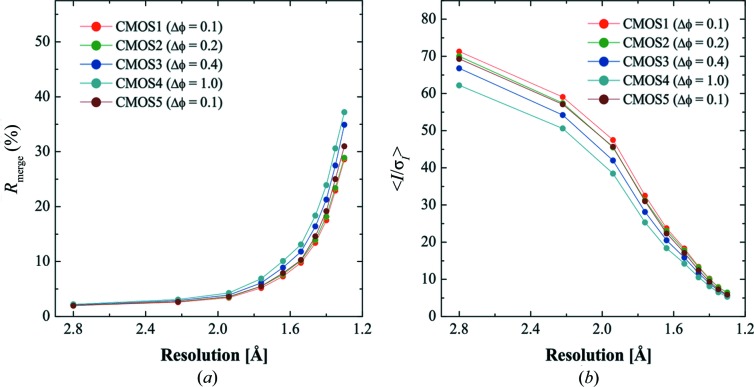
(*a*) *R*_merge_ *versus* resolution plot and (*b*) 〈*I*/σ(*I*)〉 *versus* resolution plot for five data sets collected with 0.1, 0.2, 0.4, 1.0 and 0.1° frame^−1^ by using the continuous rotation method using the CMOS detector.

Figs. 8 ▶(*a*) and 8 ▶(*b*) show the *R*_merge_ *versus* resolution plots and an 〈*I*/σ(*I*)〉 *versus* resolution plot of the data sets collected with the rotation steps of 0.1, 0.2, 0.4, 1.0 and 0.1° frame^−1^. Both statistics show that the accuracy improved as the rotation step got smaller. The *R*_merge_ values of the first and the last data sets were 28.6 and 31.0%, respectively, at the highest-resolution shell, indicating that the crystal was damaged by radiation. Therefore, the difference between the first and the other data sets included the effect of sample radiation damage as well as the effect of changing the rotation step. The *R*_merge_ values of the data sets collected with a 0.4 and 1.0° frame^−1^ step were 34.9 and 37.2%, respectively, in the highest-resolution shell, and those values were larger than that of the last data set collected with a 0.1° frame^−1^. These results show that the accuracy of data collected by the continuous rotation method using the CMOS detector was improved by using fine ϕ slicing data collection under conditions that provide strong diffraction intensities.

### 3.4. Structure determination of proteins   

**Figure 9:**
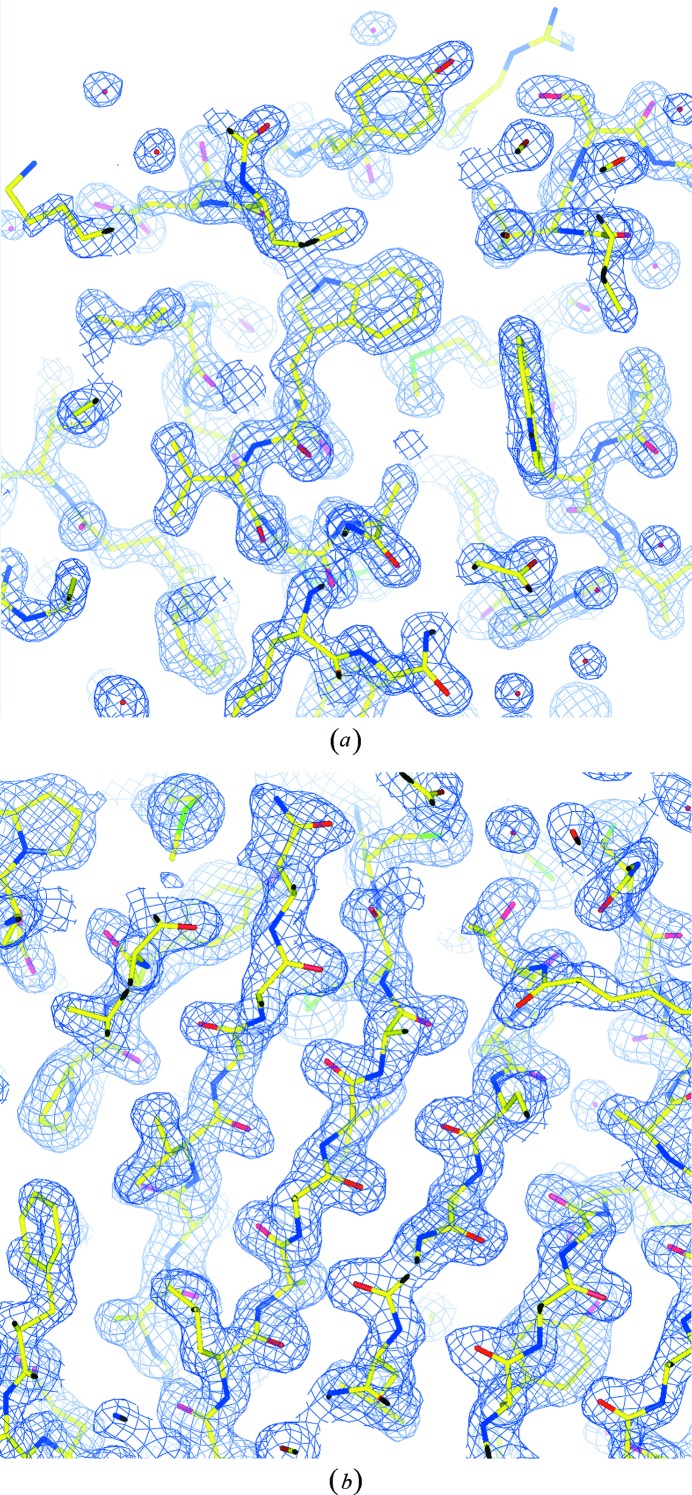
(*a*) σ_*A*_-weighted 2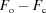 electron-density map of lysozyme Hg derivative calculated at 1.7 Å resolution. Contour level is 1.5 r.m.s. A molecular model after crystal structure refinement is superimposed on it. (*b*) Electron-density map of TTHA0593 calculated at 1.9 Å resolution. Phases were improved by density modification after SAD phasing was used. Contour level is 1.5 r.m.s. A refined structure model is also shown.

We successfully solved the structures of three proteins, a mercury derivative of hen egg white lysozyme and two selenomethionine proteins ST1066 and TTHA0593, using anomalous dispersion, and confirmed therefore the applicability of the continuous rotation method using the CMOS detector. The structures of lysozyme and ST1066 were determined by MAD phasing using the Hg and Se atoms, respectively, as anomalous scattering atoms. The structure of TTHA0593 was solved by SAD phasing using Se atoms. The initial phases of all structures were calculated by *SOLVE* (Terwilliger & Berendzen, 1999 ▶) and improved by density modification with *RESOLVE* (Terwilliger, 2000 ▶). Most residues were automatically built using *ARP*/*wARP* (Perrakis *et al.*, 1999 ▶), and the rest of the residues were manually built using *COOT* (Emsley & Cowtan, 2004 ▶). The protein structure models were refined with *REFMAC* (Murshudov *et al.*, 1997 ▶) in the *CCP4* package (Collaborative Computational Project, Number 4, 1994 ▶). The final refinement *R* factor and free *R* factor decreased to less than 21 and 25%, respectively. The data statistics and refinement statistics are summarized in Table 3 ▶.^1^ Fig. 9 ▶(*a*) shows the σ_*A*_-weighted 2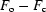 electron-density map (Read, 1986 ▶) of lysozyme after refinement, showing the clear electron density of the protein and solvent molecules. Fig. 9 ▶(*b*) is the electron-density map of TTHA0593 calculated with the phases obtained by improving the initial phases using density modification. It clearly shows the electron density of the protein main and side chains.

## 4. Discussion   

### 4.1. Characteristics of the CMOS detector   

Conventional opinion has been that the CMOS detector is not suitable for diffraction data collection because of the large dark-current noise and the narrow dynamic range. Indeed, the dark current of the detector increases in proportion to the exposure time and the effective dynamic range decreases by the dark-current effect. However, our study shows that the dark current was so stable that it could be accurately corrected by simply subtracting pre-recorded dark-current images from raw diffraction images. Moreover, a continuous readout with a high frame rate keeps the dark current at a low level. Therefore, we suppose that the dark current of the CMOS detector is not a serious problem in a high-frame-rate experiment. The evaluation of linearity showed that the linearity was maintained over an output range of 14 bits. The evaluation of the uniformity from flat-field images showed a maximum deviation of a few percent among the 18 readout segments. The data statistics of the CMOS data processed without a non-uniformity correction were comparable to the CCD data; we then processed all diffraction data sets without a non-uniformity correction. The detailed examination of the non-uniformity could be applied for improving the data statistics of the CMOS data in the next step. The intensity of the defect lines was approximated by averaging the signal intensity of neighbouring pixels. Even though this is an ineffectual estimate for the centre of the peak of diffraction spots, the intensities of pixels located at the side of a reflection profile are well approximated by this correction. This defect line correction significantly improved the data statistics. If the redundancy of the data is high, the influence of defect pixels will be small.

These results show that the CMOS detector is reasonably applicable as a two-dimensional detector for diffraction intensity measurement.

### 4.2. Feasibility and advantage of the shutterless continuous rotation method using the CMOS detector   

As the results in §3.2 show, the new continuous rotation method using the CMOS detector is applicable for protein crystallography. The diffraction data recorded by the new method were successfully processed and the data statistics of the intensity data were comparable to those obtained by the conventional oscillation method. Furthermore, the slightly better data statistics of the CMOS data compared with the conventional oscillation method show the potential of the continuous rotation method (Figs. 5 ▶ *a* and 5 ▶ *b*).

The narrow dynamic range of the CMOS detector, another potential difficulty as mentioned above, could be overcome by fine ϕ slicing. Moreover, the data statistics of the CMOS data in Fig. 7 ▶ indicate the potential for improving the data quality by the continuous rotation method with fine ϕ slicing.

The source of data improvement in fine ϕ slicing has already been discussed by Hülsen *et al.* (2006 ▶), who showed that the continuous rotation method using a PILATUS detector improved data accuracy in fine ϕ slicing mode *versus* the conventional oscillation method using a CCD detector which results in a deterioration of data accuracy. From that comparison, Hülsen *et al.* (2006 ▶) concluded that the improved accuracy of the PILATUS data was due to there being no readout noise of the PILATUS detector. On the other hand, Pflugrath (1999 ▶) pointed out that, theoretically, deviation from the proper synchronization can affect the accuracy of partially recorded reflection intensities and the influence is more serious for data collected with a small rotation step. Our result supports the latter opinion experimentally, because the data improvement is also observed in the continuous rotation method using the CMOS detector, which has some readout noise, and the comparison of data statistics among CCD data described in §3.3.1 demonstrates deterioration of the accuracy of partially recorded reflections by fine ϕ slicing. These results indicate the importance of the ‘shutterless’ mode for the fine ϕ slicing data collection.

The reduction of the overhead time between two successive diffraction images is critical when a fine ϕ step is used. If the total exposure time of one data set is kept identical, the data-collection time of the continuous rotation method using the CMOS detector does not change, regardless of the rotation step. On the other hand, the data-collection time of the oscillation method using the CCD detector increases as the oscillation step decreases. The simulated data-collection time at various rotation steps is shown in Table 4 ▶. Therefore, use of the new method has a significant advantage, in terms of the data-collection time, with regard to the use of fine ϕ slicing.

### 4.3. Future prospects for the continuous rotation method using the CMOS detector   

Our results show that the continuous rotation method using the CMOS detector C10158DK can be successfully applied to collecting diffraction data at protein crystallography beamlines. However, some improvements to the detector and data-processing software will make the new method even more useful.

One improvement to the detector is the size of the detector area; the detector area of C10158DK is 117.6 × 117.6 mm, which is smaller than that of the CCD detectors currently used in protein crystallography. The size can be increased by aligning multiple CMOS sensors in a matrix or developing a large-format CMOS sensor. The simple architecture of the CMOS detector will make this easy.

The result in §3.2.3 demonstrated the advantage of the CMOS detector for short wavelengths, which is due to the fact that the 150 µm-thickness CsI has a very high absorption of X-rays at short wavelengths (*e.g.* 97% for a wavelength of 0.8 Å). Thus, this makes the detector suitable for very short (0.5 Å) and ultra-short (0.3 Å) X-ray wavelengths, whose potential benefit was pointed out by Helliwell *et al.* (1993 ▶) for ‘ideal accuracy’ data. On the other hand, the use of C10158DK for longer wavelengths is limited, because the C10158DK uses a carbon fibre plate of 1 mm thickness as a detector window, which absorbs 58% of 1.5 Å wavelength X-rays and more for a longer wavelength. Our preliminary experiments revealed that the removal of the detector window caused a twofold improvement in the data statistics at a 1.5 Å X-ray wavelength. Modification of the window material will make the detector applicable for a very wide range of X-ray wavelengths.

The other points warranting useful effort would be to further increase sensitivity, and to reduce readout noise and dark current. As described in §3.3.1, and exemplified by Fig. 7 ▶(*d*), the smaller rotation steps for the CMOS case only improved the S/N of the strong reflections between 40 and 2 Å for lysozyme but there was no improvement for weak reflections. This is counter intuitive as there should be an improvement for weak reflections especially. This suggests the possibility of a threshold effect of the detector sensitivity disadvantaging the weaker reflection intensities. Although CCD detectors also have such a threshold effect, the accuracy of weak reflection intensities can be improved by elongating the exposure time or increasing the intensity of the incident X-ray. On the other hand, the dark current and the narrow dynamic range make it difficult for the CMOS detector to use these approaches. Therefore, an increase of sensitivity, a reduction of detector noise or a reduction of dark current would be necessary for the X-ray CMOS detector to collect weak reflection intensities more accurately.

Development of data-processing software suitable for the continuous rotation method using the CMOS detector will increase the data accuracy further. The difficulty in processing the CMOS data is due to the difference of the readout timing from the top to the bottom lines of the detector, resulting in gradual changes of the rotation angle in one image. However, no serious problem occurred in the processing of CMOS images with the *XDS*/*XSCALE* package; the reason for this may be that the mosaic spread of the crystals was larger than the maximum angular difference. The fastest angular speed of 0.25° s^−1^ used in this work makes an angular difference of 0.083°. Development of data-processing software taking account of such an angular difference will be required for smaller mosaic crystals, *e.g.* as exemplified in the finest, most perfect crystal limit of Snell *et al.* (1995 ▶).

## 5. Conclusion   

We have developed and examined the continuous rotation method using the CMOS detector. Evaluation of the basic performance of the detector showed that it is possible to use the CMOS detector for successful diffraction intensity measurement. Comparison with the conventional oscillation method using a CCD detector demonstrated that the continuous rotation method using the CMOS detector was suitable for protein crystallography with fine ϕ slicing. This method enabled rapid data collection without an overhead time and improved the data accuracy combined with fine ϕ slicing data collection. We conclude that the continuous rotation method using the CMOS detector will become a powerful tool at synchrotron protein crystallography beamlines.

## Supplementary Material

. DOI: 10.1107/S0021889809042277/he5435sup1.pdf
Data-collection condition and data statistics.

## Figures and Tables

**Table 1:** Basic specification of the C10158DK provided by Hamamatsu Photonics KK The specification of the CCD detector ADSC Q210 is also shown for comparison.

	Hamamatsu C10158DK	ADSC Q210†
Scintillator	Directly deposited CsI(Tl) (thickness 150m)	Gd_2_O_2_S:Tb
Pixel size (m)	50 50	51.27 51.27
Spatial resolution (FWHM) (m)	140‡	87
Number of pixels	2352 2352	4096 4096
Active detector area (mm)	117.6 117.6	210 210
Gain (ADUphoton^1^)	2.5 @ 0.96‡	2.4 @ 1
Gain (ephoton^1^)	168 @ 0.96	7.3 @ 1
Noise (r.m.s. e)	180	13.5
Saturation charge (e)	1.1 10^6^	2.0 10^5^
Dynamic range	6000	14100
Output data (bit)	14	16
Maximum frame rate (frames^1^)	3	

† Specification details of the ADSC Q210 were derived from the specification sheet of the manufacturer.

‡ Spatial resolution and gain of C10158DK were experimentally estimated. The spatial resolution was roughly estimated using a knife-edge slit. The shadow of the knife-edge attached on the surface of the detector was recorded and the intensity profile of the shadow was extracted. The spatial resolution was calculated by differentiating the intensity profile. The gain was estimated using a flat-field image recorded with fluorescence X-rays from bromide. The photon flux of the fluorescence X-rays was measured by an Si PIN photodiode, and then the number of ADUs generated by one X-ray photon was calculated.

**Table 2:** Statistics of data recorded with an ADSC Q210 with 1.0, 0.5 and 0.25frame^1^ oscillation steps *MOSFLM* and *SCALA* were used for processing. *R*_full_ is the *R*_merge_ of fully recorded reflections.* R*_means_full_ is the redundancy-independent *R*_merge_ of fully recorded reflections (Diederichs Karplus, 1997 ▶; Weiss Hilgenfeld, 1997 ▶; Weiss, 2001 ▶). The statistics at the highest-resolution shells are shown in parentheses.

	CCD1	CCD2	CCD3
	= 1.0 (frame^1^)	= 0.5 (frame^1^)	= 0.25 (frame^1^)
Resolution ()	251.6 (1.691.6)		
No. of reflections	110066 (15698)	109671 (15786)	106144 (14921)
Full reflection	57558	18923	0
Partial reflection	52508	90748	106144
No. of unique reflections	15051 (2205)	14988 (2179)	14799 (2127)
*R*_merge_ (%)	6.5 (33.2)	7.2 (38.8)	8.0 (46.1)
*R*_full_ (%)	5.8 (28.3)	5.1 (26.5)	
*R*_means_full_ (%)	6.4 (31.4)	6.2 (32.7)	

**Table 3:** Summary of data collection and structure determination of three proteins All data sets were collected by the continuous rotation method with the Hamamatsu C10158DK. Conditions and statistics at the absorption edge and remote wavelength data sets for lysozyme and ST1066 are given in the supplementary material for this paper. The statistics at the highest-resolution shells are shown in parentheses.

	Lysozyme	ST1066	TTHA0593
No. of residues	129	263	188
Anomalous scatteringatom (No. of atoms)	Hg (1)	Se (6)	Se (3)
Space group	*P*4_3_2_1_2	*P*6_1_22	*P*4_1_2_1_2
Cell parameter ()	*a* = *b* = 78.72, *c* = 37.09	*a* = *b* = 102.78, *c* = 105.10	*a* = *b* = 73.54, *c* = 95.55
			
Experimental conditions			
Wavelength ()	1.0075, 1.0091, 1.0259	0.9790, 0.9793, 0.9951	0.9790
Rotation step (frame^1^)	0.2	0.2	0.2
Exposure time (sframe^1^)	1	1	1
No. of images	900	900	900
			
Data statistics at peak wavelength†			
Resolution ()	501.7 (1.761.7)	502.1 (2.182.1)	501.9 (1.971.9)
No. of reflections	141537 (4172)	416512 (44083)	278320 (16536)
Completeness (%)	90.9 (62.5)	99.9 (100)	99.7 (97.4)
Redundancy	6.4 (2.8)	11.5 (11.5)	7.1 (4.2)
*R*_merge_ (%)	2.5 (8.4)	7.1 (36.7)	7.8 (34.5)
*I/*(*I*)	48.64 (12.13)	27.90 (7.73)	18.45 (3.79)
_ano_ ‡	2.52 (1.22)	1.86 (0.97)	1.31 (0.80)
			
Structure determination			
Phasing	MAD	MAD	SAD
% of amino acid residue builtby *ARP*/*wARP*	93.8	87.8	96.8
*R*_cryst_ (%)	18.7	20.6	17.6
*R*_free_ (%)	20.9	24.4	20.4

† Bijvoet pairs are merged separately.

‡ Mean anomalous difference in units of its estimated standard deviation calculated by *XSCALE*. It is defined as |*F*(+) *F*()|/**_ano_, where |*F*(+) *F*()| is the anomalous difference and **_ano_ is its estimated standard deviation.

**Table 4:** The simulated data-collection time per data set covering a 180 angular range is compared between the oscillation method using the CCD detector and the continuous rotation method using the CMOS detector The overhead time of 2.2sframe^1^, the observed value at BL38B1, is incorporated in the data-collection time of the oscillation method using the CCD detector.

				Total data-collection time (min)	
Rotation step (frame^1^)	Exposure time (sframe^1^)	No. of diffraction images	Rotation range ()	Oscillation	Continuousrotation
1.0	4.0	180	180	18.6	12
0.5	2.0	360	180	25.2	12
0.25	1.0	720	180	38.4	12
0.125	0.5	1440	180	64.8	12
0.083333	0.333333	2160	180	91.2	12

## Footnotes

1 Supplementary material for this paper is available from the IUCr electronic archives (Reference: HE5435). Services for accessing these data are described at the back of the journal.
