# Supplementary material for: Development of a shutterless continuous rotation method using an X-ray CMOS detector for protein crystallography
Source: J Appl Crystallogr. 2009 Nov 17;42(Pt 6):1165–75. doi: 10.1107/S0021889809042277 (PMC3246825; doi:10.1107/S0021889809042277)
Supplement: Supplementary file 1 [file j-42-01165-sup1.pdf]

Data collection condition and data statistics for edge and remote data of lysozyme and ST1066 crystals.

	Lysozyme		ST1066	
Experimental condition	Edge	Remote	Edge	Remote
Wavelength (Å)	1.0091	1.0259	0.9793	0.9951
Rotation step (º/frame)	0.2	0.2	0.2	0.2
Exposure time (s/frame)	1	1	1	1
Number of images	900	900	900	900
Data statistics[†]				
Resolution (Å)	50−1.7 (1.76−1.7)	50−1.7 (1.76−1.7)	50−2.1 (2.18−2.1)	50−2.1 (2.18−2.1)
Number of reflections	143204 (4098)	138750 (3337)	417369 (44039)	420100 (44026)
Completeness (%)	90.8 (61.6)	89.5 (57.6)	100 (100)	100 (100)
Redundancy	6.4 (2.7)	6.3 (2.4)	11.5 (11.5)	11.6 (11.5)
R_{merge} (%)	2.5 (8.4)	2.4 (8.5)	7.2 (38.1)	6.4 (34.7)
$\langle I/\sigma(I) \rangle$	49.58 (11.68)	50.23 (10.63)	27.64 (7.33)	32.36 (7.91)
Sig_{ano} (XSCALE) [‡]	1.96 (0.93)	1.40 (0.88)	1.72 (0.89)	0.85 (0.79)
